# Elevated cerebrospinal fluid pressure in patients with Alzheimer's disease

**DOI:** 10.1186/1743-8454-3-7

**Published:** 2006-05-31

**Authors:** Gerald Silverberg, Martha Mayo, Thomas Saul, Jere Fellmann, Dawn McGuire

**Affiliations:** 1 Department of Neurosurgery, Stanford University School of Medicine, Stanford, CA 94305, USA; 2 Genitope Corp, 525 Penobscot Drive, Redwood City, CA,94063, USA; 3 Turning Point Engineering, PO box 372 Moss Beach CA, 94038, USA; 4 Acologix Inc, 3960 Point Eden Way, Hayward, CA 94545, USA; 5 Avigen Inc, 1301 Harbor Bay Parkway, Alameda CA 94502, USA

## Abstract

**Background:**

Abnormalities in cerebrospinal fluid (CSF) production and turnover, seen in normal pressure hydrocephalus (NPH) and in Alzheimer's disease (AD), may be an important cause of amyloid retention in the brain and may relate the two diseases. There is a high incidence of AD pathology in patients being shunted for NPH, the AD-NPH syndrome. We now report elevated CSF pressure (CSFP), consistent with very early hydrocephalus, in a subset of AD patients enrolled in a clinical trial of chronic low-flow CSF drainage. Our objective was to determine the frequency of elevated CSFP in subjects meeting National Institutes of Neurological and Communicative Diseases and Stroke – Alzheimer's Disease and Related Disorders Association (NINCDS-ADRDA) criteria for AD, excluding those with signs of concomitant NPH.

**Methods:**

AD subjects by NINCDS-ADRDA criteria (n = 222), were screened by history, neurological examination, and radiographic imaging to exclude those with clinical or radiographic signs of NPH. As part of this exclusion process, opening CSFP was measured supine under general anesthesia during device implantation surgery at a controlled pCO_2 _of 40 Torr (40 mmHg).

**Results:**

Of the 222 AD subjects 181 had pressure measurements recorded. Seven subjects (3.9%) enrolled in the study had CSFP of 220 mmH_2_0 or greater, mean 249 ± 20 mmH_2_0 which was significantly higher than 103 ± 47 mmH_2_O for the AD-only group. AD-NPH patients were significantly younger and significantly less demented on the Mattis Dementia Rating Scale (MDRS).

**Conclusion:**

Of the AD subjects who were carefully screened to exclude those with clinical NPH, 4% had elevated CSFP. These subjects were presumed to have the AD-NPH syndrome and were withdrawn from the remainder of the study.

## Background

Alzheimer's disease (AD) and adult-onset, idiopathic chronic hydrocephalus, or normal pressure hydrocephalus (NPH), are age-related disorders involving various degrees of dementia. Patients with both diseases have reduced cerebrospinal fluid (CSF) production and decreased CSF turnover that may be associated with a decreased ability to clear potentially toxic metabolites, such as amyloid beta-peptides (Aβ) [1]. Several clinical series have documented the surprisingly high frequency of pathologically confirmed AD in patients meeting clinical criteria for NPH. In one series, seven of 21 NPH patients (33%) who underwent cortical biopsy at the time of ventriculo-peritoneal shunt implantation, had neuropathological changes consistent with AD [2]. In two other series, one-quarter to one-half of NPH patients had AD-type neuropathology [3,4]. In a more recent series, 23 of 55 patients (42%) biopsied at the time of shunt placement for NPH met CERAD (Consortium to establish a registry for Alzheimer's disease) criteria for AD [5], including 75% of those with severe dementia [6]. There have been no corollary studies addressing the occurrence of raised CSFP among patients with clinical AD, who have not yet developed the clinical syndrome or radiographic features of NPH.

The therapeutic objective guiding low-flow ventriculoperitoneal shunting as a treatment for AD was to improve safely the CSF turnover and clearance of metabolic by-products, such as Aβ and tau, from the brain [7]. A clinical trial designed to evaluate the safety and efficacy of low-flow shunting in subjects that met strict National Institutes of Neurological and Communicative Diseases and Stroke – Alzheimer's Disease and Related Disorders Association (NINCDS-ADRDA) criteria for probable AD [8] required that opening CSF pressure (CSFP) be measured under controlled blood pressure, oxygenation and pCO_2 _prior to shunt implantation. This allowed an estimate of the frequency of elevated CSFP and, therefore, presumed early NPH, in patients with AD, and a comparison between AD sub-groups with and without coexistent elevated CSFP. The data from the clinical trial will be the subject of a separate communication.

## Methods

The subjects in this trial were enrolled at 25 medical centers in the USA in an FDA (Federal Drug Administration) approved clinical trial of low-flow CSF shunting for AD, FDA IDE (investigational device exemption) # G 970117, protocol # 2000–01. AD subjects enrolled in the trial were in the mild to moderate phase of the disease, as indicated by a mini-mental status exam (MMSE) between 15 and 26. All subjects and their caregivers signed an informed consent as part of the experimental protocol approved by the Institutional Review Board at each center.

The 222 subjects who completed all screening and baseline assessments were randomized to receive either a functioning shunt or one that was intentionally occluded. During clinical screening and baseline psychometric testing prior to implantation of the shunt, subjects meeting strict NINCDS-ADRDA were carefully evaluated to exclude those with clinical, radiographic, or CSFP signs of NPH. By protocol these included:

1. Gait apraxia or gait ataxia, particularly one that preceded the onset of dementia. Gait apraxia (magnetic gait) was defined as the inability to initiate the lower extremity movements necessary for ambulation, not explained by leg weakness or an orthopedic condition.

2. Urinary incontinence not due to a focal, defined genito-urinary abnormality.

3. Imaging studies indicating significant enlargement of the ventricles out of proportion to the amount of cerebral atrophy. All imaging studies were independently evaluated for evidence of NPH by a neuroradiologist. Subjects with enlarged ventricles had an assessment of the degree of atrophy of the hippocampus by measuring the degree of dilation of the perihippocampal fissures (PHF), the fissures that surround the hippocampus. These include the lateral aspect of the transverse fissure, the choroidal fissure and the hippocampal fissure. Subjects with enlarged ventricles and normal or mildly dilated PHF (as seen in NPH) were excluded, whereas those with moderately or markedly dilated PHF (significant hippocampal atrophy as seen in AD) were eligible for enrollment [9].

Those subjects meeting all screening and baseline entry criteria were eligible for investigational device implantation, at which time a final entry criterion of CSFP was evaluated:

4. CSFP greater than 200 mmH_2_O was measured during initial shunt implantation surgery under general anesthesia in the standard supine position via the implanted ventricular catheter. Blood pressure and oxygenation were stable within normal parameters. The pCO_2 _was controlled at 40 Torr (end tidal CO_2 _36–37 Torr) prior to ventricular cannulation. Anesthesia consisted of propofol infusion supplemented with low dose isoflurane (<0.5%) and/or nitrous oxide (50%). As previously described, these agents do not alter CSFP [10]. Under these controlled conditions CSFP stabilized within 5–10 minutes. The opening pressure was monitored by a manometer attached to the ventricular catheter, and recorded after the CSFP had stabilized. The manometer stopcock was positioned at approximately the mid-ventricular level. If the opening CSFP was >200 mmH_2_O, generally accepted as the upper limit of normal CSFP in adults, the subject was excluded from the trial by protocol, because of probable associated early NPH. Such subjects were treated with the standard of care for NPH at the enrolling center, and at the discretion of the neurosurgeon, a conventional hydrocephalus shunt could be implanted

The subjects with elevated CSFP (AD-elevated CSFP group) were compared to the AD subjects whose CSFP was 200 mmH_2_O or less (AD-only group). Differences between the two groups in CSFP, age and in baseline Mattis Dementia Rating Scale (MDRS) scores were assessed by Student's *t *test (S_t_) and confirmed by the non-parametric Mann-Whitney two independent groups comparison (MW). The MDRS was one of the primary end-points of the study and is a comprehensive multipart test of cognitive function [11].

## Results

Of the 222 AD subjects with no clinical or radiological evidence of NPH who underwent general anesthesia for implantation of an investigational device, 181 subjects had CSFP data that could be evaluated. The remainder either did not have opening CSFP recorded for a variety of reasons, e.g. CSF was removed or lost prior to the pressure measurement, the CSFP was never recorded or the data were not available at the time of this analysis. These appeared to be random events and should not have introduced bias into the data. During the initial implantation procedure, seven of the 181 subjects (3.9%) with no clinical or radiographic signs of NPH had an opening CSFP >200 mmH_2_O (the AD-elevated CSFP group). These subjects were withdrawn from the CSF drainage trial. Therefore, they did not have an investigational device implanted nor was their follow up clinical assessment available in the case files.

**Figure 1:**
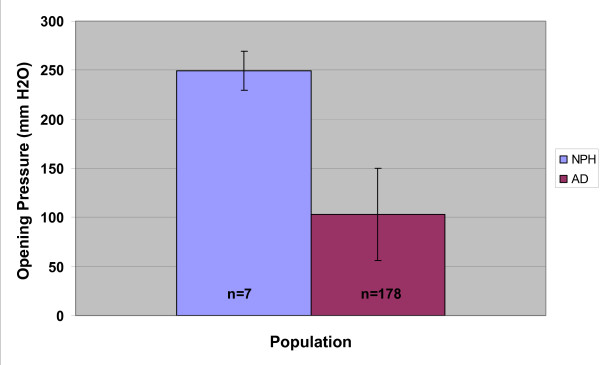
Bar graph of the mean opening pressure (CSFP) in the AD-NPH subjects, mean 249 ± 20 mmH_2_O, compared with the AD only subjects, mean 103 ± 47 mmH_2_O, S_t_*P *= 0.001, MW *P *< 0.0001.

**Figure 2:**
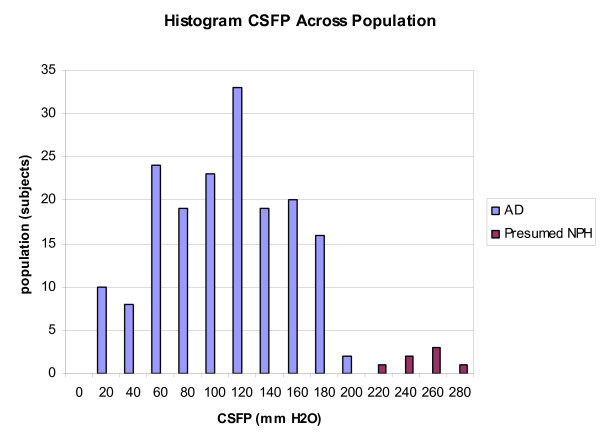
Frequency histogram showing the distribution of CSFP in all subjects. Note the two separate pressure peaks, the larger peak corresponding to the AD-only group, and a smaller peak corresponding to the AD-NPH group.

The AD-only group comprised 174 subjects whose opening CSFP at implantation surgery was 200 mmH_2_O or less. The mean age of this group was 74 ± 6 years. The mean MDRS score was 106 ± 17. Mean opening CSFP in the AD-only group was 103 ± 47 mmH_2_O. For the AD-elevated CSFP group, the mean CSFP was 249 ± 20 mmH_2_O (range 220–280 mmH_2_O), which was significantly elevated when compared to the AD-only group, S_t _*P *= 0.001, MW *P *< 0.0001 (Figure 1). The distribution of CSFP in the two populations is shown in Figure 2. The mean age of this subset of AD patients with elevated CSFP was significantly younger, 67 ± 6 years vs. 74 ± 6, *(*S_t_*P *= 0.015, MW *P *< 0.005) and the MDRS scores were significantly higher, 118 ± 6 (range 127–109) vs. 106 ± 17 (range 137–54), than the AD-only subjects, S_t_*P *= 0.001, MW *P *< 0.04). Baseline characteristics of these subjects, who we considered examples of the AD-NPH syndrome, are provided in Table 1.

**Table 1:** Baseline characteristics of the seven AD-NPH subjects. The imaging studies were read by an independent neuroradiologist. The normal MDRS score is 142–144, the normal MMSE score is 27–30. Consequently, these patients are in the mild to moderate range of AD.

Subject number	**1**	**2**	**3**	**4**	**5**	**6**	**7**
**Age**	68	66	63	71	73	56	70
**Gender**	Male	Female	Female	Male	Male	Female	Male
**Highest Education**	<8^th ^Grade	8^th ^– 12^th ^Grade	>12^th ^Grade	>12^th ^Grade	8^th ^-12^th ^Grade	8^th ^– 12^th ^Grade	8^th ^– 12^th ^Grade
**Neurological History (other than AD)**	None	None	None	None	None	Restless legs	Insomnia, headaches
**Genitourinary History**	Urinary Tract Infection, prostatitis	None	None	None	None	None	Overactive bladder, prostate hypertrophy
**Abnormal Posture**	Absent	Absent	Absent	Absent	Absent	Absent	Absent
**Brain Image Type**	MRI	MRI	MRI	CT	MRI	MRI	CT
**Markedly Enlarged Ventricles?**	No	No	No	No	No	No	No
**Other Lesions on Brain Scan**	None	Non-specific deep white matter changes	Small amt periventricular white matter changes	None	Mild changes noted on Flair image	None	None
**Gait**	Normal	Normal	Normal	Normal	Normal	Normal	Poor balance, unable to tandem gait, slow gait, no arm swinging
**MDRS**	111	123	127	120	120	118	109
**MMSE**	19	25	26	25	21	20	26
**Opening pressure**	240	235	260	260	250	220	280

## Discussion

Aging affects the CSF circulation in a number of ways [12], at least two of which influence CSF circulatory dynamics. First, there is a trend toward decreased CSF production, with a concomitant decrease in CSF turnover [13]. Second, there is an increased resistance to CSF outflow resulting in decreased CSF absorption [14]. Age-related accumulations of amyloid in the choroid plexus and meninges, fibrosis, thickening of the basement membrane, and senescent changes in the choroidal epithelium and choroidal vasculature probably play a role both in decreased CSF production and decreased CSF absorption in normal aging, in NPH and in AD [1,12,15,16]. Reduced CSF production and turnover have been demonstrated in both AD and NPH patients [1,10,17].

Growth factors that induce fibrosis may play a role in developing increased resistance to CSF absorption in some patients with AD. There appears to be an increase in the CSF concentration of transforming growth factor beta-1 (TGFβ-1) as well as an increase in brain fibroblast growth factor-2 (FGF-2) in patients with AD [18-20]. It has been shown in a transgenic mouse model, in which astrocytes over-express TGFβ-1, that hydrocephalus ensues [21]. In a rat model of FGF-2-induced hydrocephalus, both decreased CSF production and increased fibrosis of the CSF absorption pathways were demonstrated [20]. The hydrocephalus appears to be due to an increased fibrosis of the arachnoidal villi in these animal models.

The AD subjects with increased CSFP in this report cannot, strictly speaking, be called NPH, since they had neither the clinical syndrome nor the enlarged ventricles associated with NPH, otherwise they would have been excluded from enrollment. NPH itself is something of a misnomer, since patients tend to have high-normal CSF pressures with intervals of frankly elevated pressures, particularly during REM sleep [22]. Although NPH was so-named because CSFP is within the physiological range when sampled by lumbar puncture during the day [23], many studies (infusion tests and chronic CSFP monitoring) in human subjects and animals have shown that CSF dynamics in this syndrome are significantly altered [24-27]. There is increased resistance to CSF absorption and decreased compliance within the CNS. CSF abnormalities are manifest not only by abnormalities in CSF infusion tests [28], but also by nocturnal elevations in CSFP accompanied by plateau waves [24].

In an animal model of NPH, kaolin-induced hydrocephalus, CSFP is initially elevated but soon returns to the physiological range after ventricular dilatation, decreased CSF production and other compensatory events occur [29]. The individuals described herein demonstrated elevated CSFP well outside the usual CSFP associated with AD (103 ± 47 mmH_2_O) or typically found in non-demented, similarly aged controls (140 ± 60 mmH_2_O) [17]. It would be reasonable to anticipate that the patients in this study with elevated CSFP were in the earliest stages of this process at the time that their elevated pressures were discovered, and that over time they would go on to develop enlarged ventricles and clinical signs of NPH. It is also of interest that in the aged rat model of kaolin hydrocephalus, Aβ (1–40) and Aβ (1–42) accumulate in the brain compared to age-matched controls [30].

The prevalence of NPH is estimated to be one per 100,000 and that of AD is about 10% of the population over age 65 [1]. The coincidence of AD neuropathology among patients with NPH varies from 25–75% depending upon the severity of the clinical dementia. This is greater than one would anticipate if the two diseases were unrelated. Based on the current series, the approximately 4% coincidence of elevated CSFP, and presumed early hydrocephalus, among carefully selected AD patients is also greater than one would anticipate if the two diseases were unrelated.

It has been suggested that both AD and NPH are physiologically related to CSF circulatory failure [1]. If CSF failure (diminished CSF production and impaired choroid plexus transport) predominates, then AD may manifest initially. If decreased absorption, on the other hand, is the predominate manifestation of CSF circulatory failure, then NPH may emerge first. Once either disease process begins, the risk for the other disease may increase. For example, in the setting of pre-existing AD, NPH could arise with an increase in CSF outflow resistance due to amyloid deposition and fibrosis in the meninges and arachnoid granulations. Relative CSFP elevations resulting from increasing outflow resistance might lead to manifestations of NPH superimposed on AD. Similarly, among individuals with NPH at onset, amyloid clearance could be compromised by pathologic changes in outflow resistance, leading to cerebral amyloidosis and increased risk of AD. In either scenario, the two diseases could ultimately converge, resulting in an AD-NPH "hybrid".

## Conclusion

As observed in previous series, an important subgroup of patients with NPH also meets pathological criteria for AD and exhibit features of both diseases. This series represents the first set of observations documenting evidence of raised CSFP, suggesting early NPH, among carefully selected AD subjects. Despite strict clinical criteria intended to exclude NPH, elevated CSFP was observed in 3.9% of our enrolled AD study subjects. As noted, due to our strict exclusion criteria, this is likely to be a marked underestimation of the true incidence of this nosologic "hybrid", (closer to the approximately 50% incidence recorded in the NPH patients studies to date). The existence of such an AD-NPH hybrid suggests that these two diseases represent two ends of the spectrum of CSF circulatory failure.

## Abbreviations

AD: Alzheimer's disease, NPH: normal pressure hydrocephalus, CSFP: cerebrospinal fluid pressure, NINCDS-ADRDA: National Institutes of Neurologic and Communicative Diseases and Stroke – Alzheimer's Disease and Related Disorders Association, MDRS: Mattis Dementia Rating Scale, CERAD: Consortium to Establish a Registry for Alzheimer's Disease, FDA: Federal Drug Administration, IDE: Investigational device exemption, MMSE: Mini Mental Status Exam, PHF: perihippocampal fissures,

## Declaration of competing interests

The author(s) declare that they have no competing interests.

## Authors' contributions

GS described the initial hypothesis and wrote the bulk of the paper along with DM. MM, TS and JF compiled the data from the case files and did the initial statistical analysis. All authors contributed to rewriting and editing the paper. All authors have read and approved the final version of this paper.
